# Referred Cardiac Pain
^1^Read at a meeting of the Bath and Bristol Branch of the British Medical Association on March 31st, 1909.


**Published:** 1909-09

**Authors:** F. G. Thomson


"Scire est nescire, nisi id me
Scire alius sciret."

SEPTEMBER, 1909.

INDEX THROUGH

### REFERRED CARDIAC PAIN.[1]

BY

F. G. Thomson,

*B.A. (Cantab.), M.D. (Lond.).*

In certain acute forms of cardiac disease in which the pericardium is affected, it would appear that the pain experienced may be strictly local; but in the majority of cardiac lesions, more particularly in chronic disease, the pain which results is a true reflex phenomenon; and it is to this referred pain, associated specially with chronic myocardial degeneration and disease of the aortic area, that I propose to confine my remarks.

The frequency of pain in these conditions, the intense suffering which it may entail, and the dramatic suddenness with which an attack may cut short the life of a person who is apparently in reasonable health, render the subject of such clinical interest that no apology is needed for bringing it forward for consideration. The conditions more commonly associated with cardiac pain are (1) chronic myocardial degeneration, fibroid

[1] Read at a meeting of the Bath and Bristol Branch of the British Medical Association on March 31st, 1909.

or fibro-fatty ; (2) atheroma of the coronary arteries, more often than not associated with the last condition ; (3) disease of the aortic valves ; and (4) aortic aneurysm. Each of these conditions entails, at some time or another, strain, disability, and disordered function of the left ventricle.

The usual distribution of the pain of cardiac disease is over the precordia, particularly between the levels of the third and sixth ribs; along the inner side of the left arm and forearm; and in the left side of the neck. The researches of Head and Mackenzie have shown that the pain corresponds to the skin areas in the chest referable to the second, third, fourth and fifth dorsal segments, to the second and first dorsal areas in the arm and forearm, and to the second and third cervical areas in the neck.

In severe or prolonged cases the pain may affect other neighbouring somatic areas by continuity, and may spread to the right side, on which the first and second dorsal areas along the inner side of the arm are most frequently implicated.

It is important to bear in mind the exact nature of the pain under consideration, and to realise that it is not a pain, strictly speaking, in the heart, but a true reflex pain ; a pain, that is to say, of skeletal structures in certain somatic areas, which correspond to the primitive body segments from which the heart is morphologically derived, and from which its nerve supply is originally drawn.

It is interesting to note in this connection that whereas ventricular disease causes referred pain from the second to the fifth dorsal areas, the pain which accompanies certain cases of distended auricle due to mitral stenosis is referred lower down to the fifth, sixth, seventh and eighth dorsal areas. This was first pointed out by Head, and is believed to be correlated to the fact that the auricle is developed from a section of the primitive cardiac tube farther back than that which ultimately, when the tube becomes folded on itself, gives rise to the ventricle.

We are accustomed to consider the sympathetic fibres supplying the heart as efferent nerves conveying augmentor, excitatory impulses to the heart muscle. The explanation of cardiac pain now generally accepted, and which I have endeavoured to outline above, entails the supposition of afferent fibres in the sympathetic nerves, fibres which convey abnormal stimuli from the heart to the spinal cord, and there set in motion such nervous energy as gives rise to sensations of pain referred to the corresponding surface areas.

The afferent impulses leading to the painful sensations in the neck, which so frequently form part of an anginal attack, appear to be conveyed by the cardiac branches of the vagus to the medulla, whence the impulse spreads downwards to the upper part of the cervical cord.

When we consider the variety of clinical affections which may give rise to cardiac pain, we must necessarily seek for some one condition common to them all, to which we may attribute the origin of the painful sensations. There has been some difference of opinion as to what this condition really is, and different observers have held that it is due to stretching of the terminal nerve fibres in the myocardium, or to spasm or cramp of the heart muscle.

Recent advances in clinical and pathological knowledge, and especially the introduction of improved technique in the examination of the heart by graphic methods, have focussed the attention of clinicians on the autogenous functions of the cardiac muscle, the so-called primitive functions, first demonstrated experimentally by Gaskell. Gibson, His, James Mackenzie and others have attempted to correlate the symptoms of cardiac disease with abnormality or failure of the different primitive functions, and Mackenzie contends that angina and other less severe forms of cardiac pain are the direct result of impairment of the function of contractility.

The pulse characteristic of angina is a true pulsus alternans, in which every alternate beat is smaller than the one which precedes it. This indicates that the function of contractility is not adequately restored after every large beat by the time the next beat is due, a small weak beat being the result. After the relatively small expenditure of energy, due to the weaker beat, contractility is more rapidly restored, and a normal beat ensues. Thus we get large and small beats in regular and orderly alternation, a condition which is clearly distinguishable from the disordered and often tumultuous arrhythmia characteristic of nodal rhythm and extra systoles. On following Mackenzie's arguments, and analysing the sphygmographic tracings that he has been able to take during and subsequent to anginal seizures, one cannot help feeling that failure of contractility of the heart muscle affords, perhaps, the most reasonable explanation of cardiac pain. The fact that mere fatigue in a skeletal muscle does not cause pain would appear, at first sight, to invalidate the theory that anginal seizures result from impaired contractility of the heart muscle. It is, however, unsafe to draw too close a comparison between heart muscle and skeletal muscle, from which the former differs in many important respects of structure and function. Moreover, no amount of fatigue in a healthy heart gives rise to angina. Syncope occurs first, and while it is no unusual thing for temporary collapse to overcome a healthy athlete at the end of a punishing race, angina pectoris in this connection is unknown. It is a remarkable fact that a healthy heart may be worked literally to a standstill without causing anginal pain, whereas in certain conditions of myocardial degeneration the slightest exertion gives rise to a severe paroxysm. The conditions which lead to angina pectoris impose grave limitations of cardiac adaptability, and one is tempted to regard referred cardiac pain as an expression of the inability of the heart to accommodate itself to the variations of strain incidental to life.

Whatever the underlying cause may be, angina pectoris would certainly not appear to be the direct result of loss of tonicity. Not only is dilatation absent in many cases, but its occurrence, by allowing regurgitation through the auriculo-ventricular valve, may so relieve the strain on the heart muscle as to put a stop, temporarily at least, to the attacks of pain from which the patient was previously suffering. The reverse of this may also occur, and I have myself seen the relief of dilatation by cardiac tonics followed with great rapidity by anginal attacks from which the patient had not previously suffered. Moreover, though prolonged and severe exertion is usually accompanied, even in healthy athletes, by temporary dilatation, not only of the right side of the heart but also of the left, pain is not usually present, beyond a sense of fulness and tightness across the chest, in no way comparable to ordinary angina. In view of these facts, it becomes difficult to see how angina pectoris can be in any way due, as some observers have maintained, to stretching of the terminal nerve filaments in the heart muscle. On the contrary, it is precisely in those morbid conditions of the myocardium which involve loss of elasticity, and preclude ready dilatation in response to varying pressures, that angina pectoris most frequently occurs.

Coronary atheroma is so intimately associated with anginal pain that we are forced to conclude that whatever may be the ultimate exciting cause of the pain, disturbance of the coronary circulation must constitute a very important predisposing factor. Partial obliteration of the main trunk, or of different branches of the coronary arteries, leads to ischæmia of the territory supplied, and may result in failure of contractility from impaired nutrition and tardy removal of metabolic waste matter, a condition analogous to "intermittent claudication" in the limbs.

Cardiac pain varies in its intensity, within very wide limits. The agonising attacks of spasmodic pain, constituting the classical form of angina pectoris, present such distinctive features that the diagnosis is usually self-evident; and anyone who witnesses a patient during an attack—pale, sweating, immobile, hardly daring to speak or even breathe for fear of accentuating the pain or terminating his very existence—is hardly likely to mistake the condition, or underestimate the gravity of the situation. Such alarming and agonising attacks do not, however, constitute all or even the majority of the cases of referred pain which come under our notice in association with aortic or myocardial disease. In the larger number of cases the pain complained of is less severe if more persistent, and in the earlier cases is not uncommonly mistaken by the patient for a fresh manifestation of some trivial disorder such as indigestion, flatulence, rheumatism or neuralgia. In other cases the patient may complain only of some tightness across the chest, and shortness of breath on extra exertion, or on going uphill.

Inquiry into the history of such cases as present the typical features of severe angina will, in many instances, elicit the previous occurrence of attacks of evanescent and apparently trivial pain, which, if appreciated at their right value, should have served as warnings, and led, possibly, to the adoption of measures which might have deferred or even obviated the distressing and alarming attacks to which these patients have become subsequently prone. It is precisely on account of the fact that much may be done in many cases of early myocardial degeneration to postpone or mitigate its disastrous effects, and because of the important prognostic significance of the earlier and less pronounced symptoms, that special attention should be paid to these slighter attacks of referred pain. This becomes all the more important as the physical signs of the associated cardiac disability may be relatively slight and equivocal, and it is only by a careful and systematic examination of the heart and arteries that we are able to form a just estimate of the degree of myocardial degeneration present.

A hasty perfunctory examination, or failure to appreciate the significance of slight abnormalities in physical signs, may result in a very grave prognostic error. An instance of this was recently brought to my notice in the case of a strong athletic man of fifty-five, who while travelling abroad consulted a physician on account of a feeling of tightness and pain across the chest, which was attributed to indigestion, and treated as such. A week later he woke up at night with an acute paroxysm of angina, from which he died in half an hour. It is a mistake to suppose that true angina comes on only after exertion, and the statement, which occurs in certain text-books, that periodic nocturnal attacks of breast-pang are characteristic of false, as opposed to true, angina requires to be much qualified. My own experience would indicate that attacks of severe spasmodic pain, due to myocardial degeneration, are more common at night than in the day. During the last twelve months I have had under my care three cases of recurrent severe nocturnal angina, all associated with well-marked signs of myocardial disease, and all in women over seventy years of age. None of these patients was of a particularly nervous temperament, and all had outlived by many years the age at which nervous instability would be particularly likely to occur. One has since died in an attack.

The occurrence of attacks of cardiac pain during the night, when the patient is at rest, would seem to require some explanation; but assuming that the pain is due to failure of the function of contractility of the heart muscle, owing to exhaustion, we must bear in mind that the symptoms of exhaustion may not become apparent for some hours. Supposing that the crippled heart has been subjected to slight overstrain during the daytime, it is unable, like the skeletal muscles, to rest at night, and even the comparative respite obtained while the patient is lying still may not be sufficient to restore the heart's vitality, and enable the contractions to be carried out smoothly, and without undue effort. Thus the irritability engendered by the continual effort to restore dynamic equilibrium may ultimately culminate in a nerve-storm, of which the outward manifestation is an attack of angina.

The mechanical effect of flatulent distension in precipitating these attacks has probably been over-estimated. Many of the subjects of angina are not bothered with flatulence, and observation has shown that in others, in whom subsidence of an attack is accompanied by loud eructation, the wind so brought up consists of air which the patient has unconsciously swallowed during the height of the attack.

The sudden onset and spasmodic character of the pain associated with myocardial or aortic degeneration may, however, be entirely lacking. The patient may complain, instead, of a more or less continuous wearing pain either across the front of the chest, inner side of left arm, or in the neck. Such cases are not unlikely, in the absence of well-marked signs of myocardial disease, to be mistaken for intercostal rheumatism, neuralgia, or brachial neuritis. I recently had occasion to treat a patient who had been assiduously taking anti-rheumatic drugs for weeks for so-called rheumatic pain in the left arm and forearm. Inquiry elicited the occurrence of occasional slight attacks of precordial pain and faintness; and though there were at that time no obvious signs of myocardial or aortic disease beyond a ringing second sound, the history, together with a high tension pulse in a thickened artery, made it clear that the so-called rheumatism was in reality due to referred cardiac pain. Subsequent attacks of severe angina, from which the patient now suffers after undue exertion, and which have occasionally been accompanied by alarming syncopal attacks, have abundantly verified the diagnosis originally made.

Similar pain of a persistent and wearing nature may occur in the arm and neck in cases of aortic aneurysm, and if the aneurysm happen to be situated so that physical signs are slight or absent, its existence might very easily be overlooked altogether. In the case of a man under treatment some four years ago, with an aneurysm situated to the left of the sternum, the persistent pain in the left arm and neck, which he himself attributed to rheumatism, was for months the only symptom which gave him any trouble; and had it not been for the existence of hoarseness, due to paralysis of the left vocal cord, I think it is highly probable that the aneurysm, which at that period presented no other definite physical signs, would, at any rate for the time being, have escaped detection. The pain such as I have described must not be confounded with other forms of pain due to direct pressure of the aneurysm on surrounding structures. It is a true referred pain, and as its distribution corresponds so closely with that arising in lesions of the heart muscle, there is good reason to suppose that it owes its origin not to the aneurysm directly, but to the cardiac disability which the aneurysm entails.

Referred pain from the ascending aorta, which is morphologically part of the heart, and corresponds to the bulbus arteriosus of lower animals, is in practice hardly distinguishable from the pain originating from the left ventricle. Painful stimuli from the transverse aorta, which is derived from the fourth branchial arch, may, however, be referred to an area in the middle line between the sterno-mastoids and just above the sternum, an area known as Head's lower laryngeal area, and corresponding to the fourth cervical segment. This appeared to be well illustrated in the case of a prematurely aged woman of fifty-two, who consulted me a few weeks ago for persistent pain and dragging sensation in the infra-laryngeal area, above mentioned, and who was firmly convinced that she must have a growth pressing on the throat. External examination revealed no abnormality which could account for the pain, and laryngoscopic examination showed a perfectly healthy condition of the larynx and surrounding structures. Examination of the heart and arteries, however, revealed marked signs of myocardial degeneration and generalised arterio-sclerosis, and I can only account for the pain by assuming that the degenerate, and possibly dilated, transverse arch was the site of origin of the pain, which she referred so definitely to a well-marked area in the lower part of the neck.

I have quite recently seen two other atheromatous persons, whose only subjective symptom directly resulting from the condition has been pain and tenderness in the infra-laryngeal area, and I am inclined to think that on inquiry we should find this symptom present in varying degree more frequently than would at present appear to be the case.

No paper on the subject of cardiac pain would be complete without at least a passing reference to the condition described as false or pseudo-angina. The weight of evidence tends to show that to draw a hard-and-fast line between so-called true and false angina is to set up an entirely false and misleading distinction. Not only are the two extreme types linked up by a complete series of cases of graduated severity, but the evidence that cardiac disease, temporary or permanent, is absent in described cases of pseudo-angina is by no means convincing. Increased knowledge, resulting from more accurate methods of examination, has enabled us to recognise, as evidence of morbid conditions, symptoms which hitherto we have been content to regard as functional, and a careful analysis of recorded cases of so-called pseudo-angina will usually reveal the existence of signs which, though slight in degree, are similar in kind to those associated with myocardial disease.

There are three main factors which contribute to the occurrence of all forms of angina, viz. the contractile power of the cardiac muscle, the arterial pressure against which the heart has to contend, and the element of neurosis. Though the neurotic element may be, and undoubtedly is, more in evidence in certain subjects of anginal attacks, we are not justified in assuming that the nerve storm which we recognise as angina pectoris can be originated in the central nervous system apart from definite, though possibly temporary, functional disability of the heart muscle. Considerable functional abnormality may well occur apart from such gross physical changes as are capable of ocular demonstration, and may conceivably result from toxæmic influence. Excessive smoking is well recognised as a cause of cardiac pain, in addition to palpitation, arrhythmia, and other symptoms of disordered function; and it may well be that auto-intoxication from the digestive tract, or the failure of interaction of certain internal secretions, may effect such chemical changes in the heart muscle as to seriously impair its power of contraction, without producing sufficient structural alteration to be demonstrated by histological methods.

The prognosis, and to some extent the symptoms, of angina, accompanied by gross and crippling organic disease of the heart, are naturally more serious than in cases where the heart is only slightly or temporarily affected. There is very good reason, however, to consider that the essential nature of the attack is the same in both.

The treatment of cardiac pain involves the consideration of the treatment of the various cardiac conditions which lead up to it. When organic changes in the myocardium are well advanced, we can do little to prevent the attacks beyond insisting on physical rest and avoidance of worry or excitement. In cases where disease is less advanced, and the myocardium possesses a greater degree of recuperative power, the institution of a healthy, quiet regime, with strict attention to diet, and the adoption of such medicinal and other measures as will ensure free elimination, diminution of intestinal intoxication, and reduction of arterial tone, may do much to relieve pain and promote the general welfare of the patient. In cases where the neurotic element predominates, removal of worry, avoidance of excitement, and abstention from fatigue, are first essentials, and, together with the administration of sedatives—especially bromides—and tonics, such as iron or arsenic, are the measures best calculated to relieve the condition.

The treatment of an actual attack of angina demands three considerations: (1) Rest, to promote restoration of heart power; (2) vaso-dilators, to diminish cardiac strain; and (3) morphia, to relieve the pain when this is not achieved by rest and amyl nitrite, or nitro-glycerine. In cases of severe spasmodic pain in middle-aged people, amyl nitrite, by lowering arterial tension, may provide instant relief; but in those cases of advanced fibroid degeneration in old people in which severe, prolonged, frequently-recurring attacks of cardiac pain render life a burden, the only drug which seems to give relief is morphia, or failing that the administration of chloroform. In either case the relief is only temporary, and the repeated administration of one or other of these drugs may be necessary to render bearable the closing stages of a distressing and painful condition.

